# Septoplasty in children: problem or solution?

**DOI:** 10.5935/1808-8694.20130073

**Published:** 2015-10-08

**Authors:** Octavius Piltcher

**Affiliations:** Assistant Professor - Department of Ophthalmology and Otorhinolaryngology - Federal University of Rio Grande do Sul (UFRGS).

For those who follow the history of septoplasty in children, reading the paper “The impact of Metzenbaum septoplasty on nasal and facial growth in children”, published in this issue (pg. 454), seems to be one more whiff of evidence in the slow clearing of this dogma: nasal surgery in the pediatric population. The “puff” should not be interpreted unflattering but realistic, as the real “storm” of evidence required still collides with our inability to design studies more suitable for this purpose.

But the question is: is such design feasible? Will it ever be feasible? Unfortunately, since we are dealing with the evaluation of a treatment, i.e., a surgical procedure, ideally we should have a randomized trial among children with equal septal deviation, ideally identical twins, in which both would be treated under anesthesia with subperichondrial detachment, but the cartilage would be removed in only one of them and the incisions, if needed, would be carried out according to the Metzenbaum technique.

It might even be feasible in experimental animal models; however, there will still be phylogenetic questions concerning the application of such findings in another species. If this argument is true and valid, it would be reasonable to also question with what quality of evidence childhood septoplasty became such an inhospitable topic.

Despite the need for a constant critical follow up of the literature, practice requires us to develop a capacity to absorb the information available so that, associated with one's personal experience, we can offer the best treatment option to our patients.

The discussion involving septoplasty in children has always focused on the possible negative consequences of the procedure on the nasal and craniofacial growth of the patients. Interesting is that such a perspective - for many years discussed under this light - now probably driven by the development of many assessment centers for mouth breathing children and the attainment of cosmetic and functional results through appropriate interventions ever earlier in children with various craniofacial malformations, brings up an insight under a different light. Septoplasty, with some technical restrictions, not only does not harm craniofacial growth, but it can improve such development. Is having a better nasal airflow and its consequence on facial bone growth vectors more important than the preservation of an intact quadrangular cartilage in children?

While evidence is emerging and being discussed, I could not finish without stating my personal opinion on this, and it depends, first and foremost, on a correct diagnosis. It may seem simple, but in adults, whom are able to clearly verbalize their complaints, we do not have diagnostic tools that allow us to determine exactly who are the patients to benefit from surgical treatments and who will not, let alone in children.

Well, I consider it a proper diagnosis when faced with a child with clinical nasal obstruction, in which the physical examination identifies septal deviation that significantly compromises nasal airflow. After this step, even before a family full of expectations for resolving the problem as soon as possible, i.e. surgical treatment, the risk of any negative change in facial growth pattern for this option must be properly discussed with the family.

On the other hand, there are two important aspects favoring surgery that must be discussed. The first is the quality of life obtained by the reestablishment of a suitable nasal airflow. This aspect alone has a greater effect on me as a doctor when making a decision. The second aspect is the possible positive impact on the craniofacial growth of these patients. By talking with the different professionals working with mouth breathing children (ENT, maxillofacial surgeons, dentists and speech therapists, for instance) the impression I have is that these aspects must be and are being prevalent in decision making. However, we must be clear, especially in the editorial of a scientific journal, that the level of evidence reached in this context is not yet satisfactory and definitive.

